# Is innovation in surgery less than ideal? A case study of acellular dermal matrix assisted prosthetic breast reconstruction

**DOI:** 10.1186/1745-6215-14-S1-P1

**Published:** 2013-11-29

**Authors:** Shelley Potter, Danielle Browning, Jelena Savovic, Rob Warr, Simon Cawthorn, Jane Blazeby

**Affiliations:** 1 Centre for Surgical Research, University of Bristol, Bristol, UK; 2 Breast Care Centre, North Bristol NHS Trust, Bristol, UK; 3 Department of Plastic Surgery, North Bristol NHS Trust, Bristol, UK; 4 Division of Surgery, Head and Neck, University Hospitals Bristol NHS Foundation Trust, Bristol, UK; 5 Department of Surgery, Royal United Hospital Bath, Bath, UK

## Introduction

The introduction of innovative procedures requires appropriate evaluation. IDEAL recommendations propose four stages of evaluation, (**I**dea-**D**evelopment-**E**valuation-**A**ssessment-**L**ong-term study). The aim of this study was to review the introduction of an innovative surgical technique according to this framework.

## Methods

Literature searches identified articles published between 2000 and 2012 reporting acellular dermal matrix-assisted prosthetic breast reconstruction (ADMPBR). Studies were classified by IDEAL-stage as A)descriptive (IDEAL-1/2a) reporting the feasibility or development of ADMPBR or B)comparative (IDEAL-2b/3(RCTs) comparing ADMPBR with standard techniques. IDEAL study designs reported before and after 2008/9 were examined to explore progression of study design over time.

## Results

Of 236 abstracts, 50 papers reporting data on 3,648 patients were included. 24 (48.0%) were IDEAL-1/2a;25 (50.0%) IDEAL-2b and 1 (2%) IDEAL-3. IDEAL-2b-studies significantly increased from period-1 (2005-2008) to period-2 (2009-2012)(n=1 to n=24,p<0.01). The number of IDEAL-1/2a-studies published annually remained constant (n=2-4). Almost all IDEAL-1/2a studies (n=20,87.0%) provided comprehensive descriptions of surgical technique, but less than half (n=11) reported patient-selection criteria and only 25% documented seeking patient consent IDEAL-2b-studies were significantly larger than IDEAL-1/2a-studies (IDEAL-1/2a-median= 39, inter-quartile range-20-65 vs.IDEAL-2b-median=73, inter-quartile range-36-186,p<0.01,Median-test) and more likely to report combined results from groups of surgeons (n=10 vs.n=5;p=0.06). Short-term complication reporting was more comprehensive in IDEAL-2b-studies but there were no differences in the reporting of histological or technical details across groups and IDEAL-1/2a-studies were significantly more likely to report long-term (p=0.03), patient-reported (p<0.01) and cosmetic outcomes (p=0.05).

## Conclusions

The introduction of ADMPBR does not consider previous evidence and comparative studies are lacking. Well-designed and conducted studies are needed to appropriately evaluate novel surgical innovations to establish standards of care, protect patients and surgeons.

